# Rate of neuropathic progression in hereditary transthyretin amyloidosis with polyneuropathy and other peripheral neuropathies: a systematic review and meta-analysis

**DOI:** 10.1186/s12883-021-02094-y

**Published:** 2021-02-12

**Authors:** Xiaochen Lin, Aaron Yarlas, Montserrat Vera-Llonch, Nishtha Baranwal, Josh Biber, Duncan Brown, Braden Vogt, Chafic Karam

**Affiliations:** 1 QualityMetric Incorporated, LLC, Johnston, RI USA; 2 Akcea Therapeutics, Cambridge, MA USA; 3 Department of Neurology, Oregon Health & Science University, Portland, OR USA; 4 Department of Neurology, University of Pennsylvania, Philadelphia, PA USA

**Keywords:** Hereditary transthyretin amyloidosis with polyneuropathy, Neuropathy impairment score, Meta-analysis, Peripheral neuropathy, Neuropathic progression

## Abstract

**Background:**

We aimed to compare neuropathic progression rate between hereditary transthyretin amyloidosis with polyneuropathy (ATTRv-PN) and other peripheral neuropathies, including diabetic peripheral neuropathy (DPN) and Charcot-Marie-Tooth disease (CMT).

**Methods:**

Literature searches identified studies reporting neuropathic progression, measured by Neuropathy Impairment Score (NIS) or NIS-Lower Limbs (NIS-LL). Our study also included unpublished data from a clinical registry of patients who were diagnosed with different peripheral neuropathies and seen at the Oregon Health & Science University (OHSU) during 2016–2020. Meta-analysis and meta-regression models examined and compared annual progression rates, calculated from extracted data, between studies of ATTRv-PN and other peripheral neuropathies.

**Results:**

Data were synthesized from 15 studies in which NIS and/or NIS-LL total scores were assessed at least twice, with ≥12 weeks between assessments, among untreated patients with ATTRv-PN or other peripheral neuropathies. Meta-analysis models yielded that the annual progression rate in NIS total scores was significantly different from zero for studies in ATTRv-PN and CMT (11.77 and 1.41; both *P* < 0.001), but not DPN (− 1.96; *P* = 0.147). Meta-regression models showed significantly faster annual progression in studies in ATTRv-PN, which statistically exceeded that in other peripheral neuropathies by 12.45 points/year for NIS, and 6.96 for NIS-LL (both *P* < 0.001).

**Conclusions:**

Peripheral nervous function deteriorates more rapidly in patients with ATTRv-PN than for other peripheral neuropathies. These findings may improve understanding of the natural history of neuropathy in ATTRv-PN, facilitate early diagnosis, and guide the development of assessment tools and therapies specifically targeting neuropathic progression in this debilitating disease.

**Supplementary Information:**

The online version contains supplementary material available at 10.1186/s12883-021-02094-y.

## Background

Hereditary transthyretin amyloidosis with polyneuropathy (ATTRv-PN) is a rare disease characterized by progressive sensory, motor, and autonomic neuropathy [1]. Patients with ATTRv-PN have a life expectancy of 5 to 15 years from symptom onset and experience considerable burden of illness [2, 3] that increases with disease progression [4, 5]. Recently approved treatments for ATTRv-PN show evidence of slowing neuropathic progression [6–9], with earlier treatment initiation predicting better prognosis [10]. Hence, earlier recognition of this disease may lead to earlier diagnosis and improved disease management.

Patients with ATTRv-PN experience rapid neuropathic progression [11]. Indirect comparison suggests a substantially faster rate of progression in patients with ATTRv-PN than in patients with other peripheral neuropathies, such as diabetic peripheral neuropathy (DPN) [12]. However, there have been no direct empirical comparisons of the rates of neuropathic progression between patients with ATTRv-PN and patients with other peripheral neuropathies. Furthermore, ATTRv-PN may be difficult to differentiate from idiopathic peripheral neuropathy (IPN) or DPN in early stages of disease [13–16].

To fill this gap, we conducted a systematic literature review and meta-analysis to identify evidence from the literature on neuropathic progression for different peripheral neuropathies, and to quantify and compare the rate of neuropathic progression in ATTRv-PN with that of other peripheral neuropathies. The objective of this study, then, was to better understand the natural history of peripheral neuropathy across different conditions, and to provide an interpretive context for the time-course of neuropathic degeneration in ATTRv-PN that could potentially reduce misdiagnosis of this condition.

## Methods

### Measure of peripheral neuropathy: neuropathic impairment score (NIS)

The NIS is a widely used, clinician-rated measure that captures the symptoms and signs of neuropathic progression across different disease types that manifest in peripheral neuropathy. The NIS was originally developed for assessing neuropathic progression and response to treatment in DPN, but this instrument and its related measures have also been utilized in other types of peripheral neuropathy [7, 17–19]. In particular, variations of the measure have been used as primary endpoint measures in pivotal RCTs of patients with ATTRv-PN [6, 9], because of its high sensitivity in tracking progression of neuropathy over relatively short time periods [12]. Therefore, the NIS can be used to compare the rate of neuropathic progression between ATTRv-PN and other disease types manifesting in peripheral neuropathy.

The NIS uses a composite clinical scoring system, with a trained neurologist evaluating muscle weakness, reflexes, and sensation at specific sites bilaterally [20]. NIS total score is graded on a scale of 0–244, with higher scores indicating greater impairment [18]. The NIS-LL is a subset of NIS items specific to neuropathy in the lower limbs. NIS-LL total score ranges from 0 to 88, with higher scores indicating greater impairment. Other variants of the NIS (e.g., modified NIS + 7 [mNIS + 7] scale) have been used in studies of patients with ATTRv-PN [6, 9].

### Literature search

All methods used for the literature review, including choice of databases and search engines, search strings, and eligibility criteria for study selection, were documented in a literature search protocol that was finalized prior to conducting the search (available upon request to the corresponding author). The literature search was conducted using multiple sources.

#### PubMed

Two search strings were entered separately into the PubMed search engine. *String A* aimed to identify the literature reporting on the change-over-time in NIS-related measures among untreated patients with any health condition manifesting in peripheral neuropathy, while *String B* aimed to identify the literature related to peripheral neuropathy specifically for patients with ATTRv-PN, to better ensure that studies of these latter patients were retrieved. *String B* included a filter for articles published in 2017 or later, as any relevant studies published prior to 2017 were already retrieved using a standardized literature review (SLR) commissioned by Akcea Therapeutics in May, 2018 on the topic of ATTRv, for which the report accessible to researchers. The search strategy used for this previously SLR is included in Supplementary Materials. *String A* and *String B* were combined using the “OR” operator to avoid retrieval of duplicate records.

- *String A*: “neuropathy impairment score” OR (“NIS” AND neuropath*) OR (“impairment score” AND neuropath*) OR (“NIS” AND “lower limb”) OR “NIS-LL” OR (“NIS + 7” OR “mNIS+ 7”)
- *String B*: (hereditary transthyretin amyloidosis OR “hATTR” OR “hereditary ATTR”) AND (polyneuropath* OR (peripher* AND neuropath*)) AND (“2017/01/01”[Date - Publication]: “3000”[Date - Publication])

#### ClinicalTrials.gov

The following terms were entered or selected into pre-specified filters in the ClinicalTrials.gov search engine.

- Condition or disease: <*blank*>
- Other terms: neuropathy impairment score OR (NIS AND neuropathy) OR (NIS-LL)
- Study type: All Studies
- Study Results: All Studies
- Recruitment Status: Completed AND Active, not recruiting

#### Additional sources

To identify potentially relevant studies not captured by database searches, investigators also manually reviewed records from the following sources: reference lists of records selected for full-text review and from relevant review articles retrieved in the database search; references in Akcea Therapeutics’ SLR report mentioned above, and a bibliography provided to the researchers by Dr. Peter J. Dyck, the developer of the NIS (personal communication). In addition to studies in the published literature, the current analysis also included unpublished data from a clinical registry study of patients with ATTRv-PN, DPN, CMT, or IPN at the Oregon Health & Science University (OHSU) between 2016 and 2020 (Karam Chafic: A registry study of patients with different peripheral neuropathies, unpublished). For this study, retrospective chart reviews were performed to NIS and NIS-LL scores for ten patients within each condition. This registry study was approved by the OHSU Institutional Review Board (IRB).

### Study selection

The following inclusion criteria were used to select records identified in all searches:

- Record available in English
- Record was not a review, case report, study protocol, letter, or editorial
- Study sample included human patients ≥18 years
- Study sample included patients with a health condition that manifests in peripheral neuropathy
- Study sample included patients not receiving active treatment for peripheral neuropathy (including patients receiving placebo ‘treatment’) during the assessment period
- NIS and/or NIS-related measures was assessed at two or more times, with a duration ≥12 weeks in between the initial and final assessments
- The record reported extractable data for computing rate of change in NIS and/or NIS-related measures

All records identified from the literature search were screened in two rounds, the first by the title and abstract, and the second by the full text. Within each round, each record was screened independently by at least two reviewers (from among co-authors XL, AY, NB, and JB). Any disagreements among the subset of reviewers regarding study inclusion were resolved through consensus-based discussion among all reviewers.

### Risk of Bias assessment

Methodological risk of bias for each study selected for inclusion in the data synthesis was assessed using tools recommended by the Cochrane Collaboration: the Cochrane Collaboration’s tool for RCTs [21], and the Newcastle-Ottawa Scale for non-interventional studies [22]. The Cochrane Collaboration’s tool assesses several study attributes, including random sequence generation, allocation concealment, blinding of participants and personnel, blinding of outcome assessment, incomplete outcome data, selective reporting of outcomes, and other sources of bias. For each RCT, the study-level risk of bias was considered low if at least four out of six attributes were rated as low risk of bias. The Newcastle-Ottawa Scale assigns a certain number of “stars” representing the potential for bias in multiple aspects within three attributes of each study, with fewer stars indicating higher risk of bias: selection of groups (0–4 stars), comparability of groups (0–2 stars), and the determination of outcomes (0–3 stars). Number of stars are summed across attributes to produce an overall assessment of study bias, with a sum < 7 stars indicating a study with high risk of bias.

### Data extraction

Relevant data were extracted from each study to estimate the change in scores from NIS-related measures over specified periods of time for each relevant sample. Numeric data were extracted directly from each record. Data reported only graphically in a report (e.g., in a figure) were estimated using WebPlotDigitizer software (https://apps.automeris.io/wpd/), which has demonstrated high levels of inter-coder reliability and validity when used for this purpose [23]. Two researchers (co-authors XL and AY) independently estimated numeric values from reported graphics using WebPlotDigitizer; estimated values converted from spatial distances were compared between the two coders to ensure similarity, and then were averaged.

Available data on change in NIS-related measures were extracted from each record, including: 1) mean changes of NIS-related measures from initial assessment and the corresponding standard deviations (SDs) or standard errors (SEs), 2) mean scores of NIS-related measures at initial assessment and follow-up visits with the corresponding SDs or SEs, and 3) rate of change in NIS-related measures. These data were extracted for all available NIS-related measures (including total scores and domain scores), and were entered into an Excel spreadsheet separately for each instrument/domain and for each follow-up assessment of each individual study.

If the mean scores from NIS-related measures at each assessment were directly reported but the mean changes from initial assessment to follow-up assessments were not, then the mean changes were calculated in Excel by subtracting the mean scores at the initial assessment from those at each follow-up assessment. If not reported, the corresponding SD for change scores was imputed based on the variances in scores at each of the two assessments and the covariance in scores between the two assessments, assuming a conservative correlation coefficient of 0.5 [24]. The mean difference (MD) in score corresponding to a one-year period of time (i.e., the mean annual rate of change, in points/year), if not directly reported in a record, was calculated for each follow-up assessment by dividing the mean change from initial assessment of each NIS-related measure to each follow-up assessment by the duration between initial assessment and follow-up assessments in yearly units. Excel formulas were used for all conversions involving extracted data.

Aside from data on NIS-related measures, data on sample and study characteristics were also extracted from each included study, including mean (SD) age at initial assessment, gender distribution, study type (RCT or non-interventional study), sample size of the analytic sample at each assessment, and duration (in weeks) between initial and each follow-up assessment.

### Data synthesis

All statistical analyses were performed with Stata statistical software version 14.0 (Stata Corp). Two-sided *P* ≤ 0.05 was used as the significance level.

#### Primary analysis

Since the duration between initial and follow-up assessments varied across studies, the outcome variable for all models in the primary analysis was annual rate of change as calculated using NIS-related scores from initial to final assessment from each study. For studies that reported multiple follow-up assessments, only the annual rate of change calculated using the final assessment was included in the primary analysis because the rate of changes based on any earlier assessments were nested within (and thus not independent from) the rate of change at the final assessment. For studies with a single follow-up assessment, that assessment was considered the final study assessment. Annual rate of change when calculated using non-final follow-up assessments were examined in sensitivity analyses described below.

To synthesize evidence across multiple studies, mean annual rate of change and the corresponding SE from each study were used to compute the weighted mean annual rate of change and the corresponding 95% confidence intervals (CIs) across all studies within each disease type. The DerSimonian and Laird random-effects model was used for the analysis, with the underlying assumption that data comes from varied populations with different distributions, given the potential heterogeneity across studies [25]. Between-study heterogeneity was examined using the *I*^2^ statistic. *I*^2^ ≈ 25 % , 50 % , 75% is suggestive, respectively, of low, medium, and high heterogeneity [26, 27]. Publication bias was not examined, following the recommendation of the Cochrane Collaboration that tests for funnel plot asymmetry should only be used when there are at least 10 studies included in the meta-analysis [24], a criterion that was not met for any of the meta-analysis models tested in the current research.

Meta-regression was used to evaluate the impact of a moderator variable – disease type, with studies coded as including either 1) patients with ATTRv-PN, or 2) patients with other peripheral neuropathies – on annual rate of change in NIS-related measures in the primary analysis. This technique affords testing whether the annual rate of change in NIS-related scores statistically differed between studies of patients with ATTRv-PN and studies of patients with other peripheral neuropathies. Additionally, a second meta-regression model was fitted with two moderator variables – disease type (coded as in the previous model) and score at initial assessment on the NIS-related measure – to test for differences in annual rate of change in NIS-related scores between disease types when controlling for patients’ severity of neurological impairment at initial assessment. The annual rate of change score was included in meta-regression models as the dependent variable, with a dichotomous variable for disease type (in both meta-regression models) and a continuous variable for initial assessment score (in the second meta-regression model) included as the independent variable(s). For each model of each NIS-related measure, the estimated coefficient for weighted MD in annual rate of change, the corresponding 95% CI for the estimate, and the *P* value for differences between disease types are reported.

#### Sensitivity analyses

To ensure the robustness of the primary findings, three sets of sensitivity analyses were performed: 1) the outcomes of models were calculated as the annual rate of change in NIS-related measures using change scores from initial assessment to the second-to-last assessment, rather than the last assessment as in the primary analysis, from studies with multiple follow-up assessments (for studies with only a single follow-up assessment, data from that assessment was included) (Sensitivity Analysis I); 2) models excluded studies with a sample size < 20 patients (Sensitivity Analysis II); and 3) models excluded studies rated as high risk of bias (Sensitivity Analysis III). Specifications for meta-analysis and meta-regression models for all sensitivity analyses were identical to those described for the primary analysis.

## Results

### Overview of selected studies

**Fig. 1:**
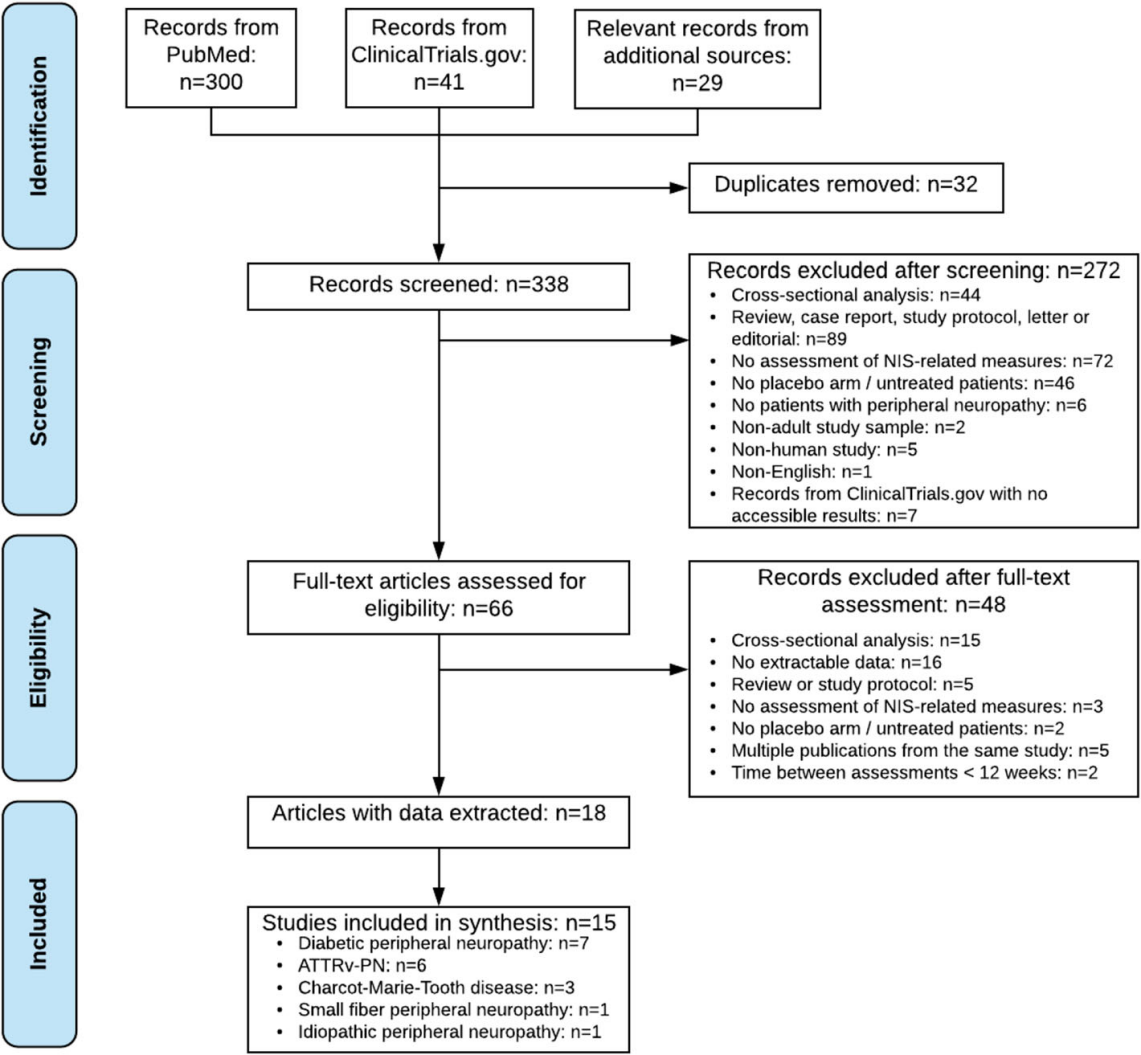
PRISMA Flowchart of Study Section. Abbreviations: ATTRv-PN, hereditary transthyretin amyloidosis with polyneuropathy; NIS, Neuropathy Impairment Score; PRISMA, Preferred Reporting Items for Systematic Reviews and Meta-Analyses

Figure 1 shows a Preferred Reporting Items for Systematic Reviews and Meta-Analyses (PRISMA) diagram that details the process of study selection and reasons for exclusion. As shown in Fig. 1, 15 studies were included in the data synthesis after removing duplicated records, two rounds of screening, and inspection of the extracted data [6–8, 19, 28–37] (Karam Chafic: A registry study of patients with different peripheral neuropathies, unpublished). WebPlotDigitizer was used to extract data that were only reported graphically from two records [8, 28].

Amongst the 15 studies, four were non-interventional studies [30–32] (Karam Chafic: A registry study of patients with different peripheral neuropathies, unpublished) and the remaining 11 were RCTs [6–8, 19, 28, 29, 33–37]. Eleven studies reported the neuropathic progression measured by the total NIS score, and nine studies reported the change of the total NIS-LL score over time. For all other NIS-related measures, such as the NIS with 7 nerve tests (NIS + 7) and the modified NIS + 7 (mNIS+ 7), as well as for specific domains of the NIS or NIS-LL, available data could only be synthesized for studies within a single disease type, thereby not affording comparisons of annual rate of changes across disease types. Thus, in all analytic models, only mean annual rate of change in NIS and NIS-LL total scores were analyzed.

Characteristics of the included studies are summarized in Table 1; statistics for the initial assessment scores of NIS and NIS-LL and the annual rate of change from initial assessment to each follow-up assessment for each study are summarized in Table 2. The mean age ranged from 33 to 69 years among the included studies; the percentage of male participants ranged from 24 to 90%; the follow-up duration ranged from 160 days to four years, except three studies with varying follow-up durations for individual patients [31, 32] (Karam Chafic: A registry study of patients with different peripheral neuropathies, unpublished). Study characteristics and initial assessment scores are also summarized by disease type for NIS and NIS-LL, respectively (Table 3).

**Table 1:** Characteristics of Selected Studies

First Author /Principal Investigator	Year	Study Name/Identifier	Study Type	Disease Type	Age: Mean (SD)	Male Gender: N (%)
Benson [6]	2018	NEURO-TTR: NCT01737398	RCT	ATTRv-PN	60 (14)	41 (68%)
Berk [7]	2013	NA	RCT	ATTRv-PN	59 (12)	44 (67%)
Coelho [8]	2012	FX005: NCT00409175	RCT	ATTRv-PN	38 (13)	26 (43%)
Hernandez-Ojeda [28]	2012	NA	RCT	DPN	57 (9)	6 (24%)
Hor [29]	2018	NCT01973400	RCT	DPN	57 (9)	63 (42%)
Karam^a^	2020	NA	Non-interventional study	CMT	51 (13)	6 (50%)
				DPN	65 (11)	4 (36%)
				ATTRv-PN	67 (7)	9 (90%)
				IPN	66 (13)	7 (58%)
Luigetti [30]	2018	NA	Non-interventional study	ATTRv-PN	69 (9)	15 (83%)
Mundayat [31]	2018	THAOS	Non-interventional study	ATTRv-PN	41 (9)	NA
Sahenk [19]	2005	NA	RCT	CMT	33 (5)	3 (75%)
Shy [32]	2008	NA	Non-interventional study	CMT	40 (19)	30 (42%)
Tesfaye [33]	2007	Rochester Diabetic Neuropathy Study: NCT00044408 and NCT00044395	RCT	DPN	48 (9)	115 (44%)
Windebank [34]	2004	NA	RCT	Small fiber peripheral neuropathy	62 (11)	NA
Ziegler [35]	1999	ALADIN 3	RCT	DPN	57 (6)	83 (50%)
Ziegler [37]	2009	AV-007-IM: NCT00483730	RCT	DPN	56 (6)	77 (27%)
Ziegler [36]	2011	NATHAN 1: NCT00977483	RCT	DPN	54 (8)	150 (67%)

*Abbreviations*: *CMT* Charcot-Marie-Tooth disease, *DPN* diabetic peripheral neuropathy, *ATTRv-PN* hereditary transthyretin amyloidosis with polyneuropathy, *NA* not applicable, *RCT* randomized controlled trial, *SD* standard deviation

^a^Unpublished dataset

**Table 2:** Initial Assessment Scores and Mean Annual Rate of Change in NIS and NIS-LL Total Scores from Initial Assessment to Each Follow-up Assessment in Eligible Studies

First Author/Principal Investigator	Year	Disease Type	Measure	Initial Assessment Scores			Rate of Change from Initial Assessment			
				N	Mean	SD	Time Point	N	Mean	SE
Benson^a^ [6]	2018	ATTRv-PN	NIS	59	43.40	24.66	35 weeks	56	7.98	1.32
							66 weeks	52	18.65	1.76
			NIS-LL	59	28.72	15.99	35 weeks	56	3.66	0.76
							66 weeks	52	9.61	1.00
Berk [7]	2013	ATTRv-PN	NIS	66	45.40	46.30	1 year	37	10.10	1.63
							2 years	28	23.20	2.73
			NIS-LL	66	27.20	24.50	1 year	37	6.00	1.10
							2 years	28	12.10	1.63
Coelho [8]	2012	ATTRv-PN	NIS-LL	61	11.40	13.54	6 months	57	2.05	0.66
							12 months	50	4.72	0.77
							18 months	47	5.83	0.92
Hernandez-Ojeda^b^ [28]	2012	DPN	NIS	25	7.40	4.70	12 weeks	25	0.70	1.15
Hor [29]	2018	DPN	NIS	150	15.80	8.41	6 months	150	−3.30	0.54
							12 months	150	−3.90	0.61
Karam^c^	2020	CMT	NIS	12	59.79	24.22	Annual rate of change from patients with various follow-up durations	12	1.94	1.34
			NIS-LL	12	35.88	12.83		12	1.89	0.92
		DPN	NIS	11	12.82	8.68		11	1.19	0.72
			NIS-LL	11	8.27	4.31		11	1.09	0.57
		ATTRv-PN	NIS	10	29.15	29.91		10	18.31	4.41
			NIS-LL	10	15.95	14.00		10	9.35	1.70
		IPN	NIS	12	34.35	22.78		12	0.42	0.99
			NIS-LL	12	24.25	14.06		12	0.43	0.39
Luigetti [30]	2018	ATTRv-PN	NIS	18	69.61	49.73	12 months	13	6.50	1.38
							24 months	3	8.33	5.04
Mundayat [31]	2018	ATTRv-PN	NIS-LL	167	7.57	7.29	Annual rate of change over 2 years	167	2.94	0.37
Sahenk^b^ [19]	2005	CMT	NIS	4	17.75	9.73	24 weeks	4	1.25	5.20
Shy [32]	2008	CMT	NIS	72	62.50	19.25	Annual rate of change from patients with various follow-up durations	72	1.37	0.38
Tesfaye [33]	2007	DPN	NIS-LL	262	6.95	5.00	1 year	211	0.63	0.23
Windebank [34]	2004	Small fiber peripheral neuropathy	NIS	NA	NA	NA	6 months	18	−0.40	1.15
Ziegler [35]	1999	DPN	NIS	165	14.00	10.40	7 months	124	−4.37	0.83
			NIS-LL	165	11.00	7.30	7 months	125	−3.37	0.54
Ziegler [37]	2009	DPN	NIS-LL	286	8.80	7.30	160 days	286	−3.70	0.30
Ziegler [36]	2011	DPN	NIS	224	12.20	7.80	2 years	207	0.12	0.43
							4 years	207	0.61	0.46
			NIS-LL	224	9.50	5.30	2 years	207	0.03	0.29
							4 years	207	0.43	0.31

^a^Initial assessment scores and change scores from the NEURO-TTR trial were extracted from clinical study report (CSR) corresponding to this study, which was made available to researchers by Akcea Therapeutics, Inc.

^b^Changes scores and the corresponding SEs were calculated from the scores at the initial assessment and each follow-up assessment

^c^Unpublished dataset

*Abbreviations*: *CMT* Charcot-Marie-Tooth disease, *DPN* diabetic peripheral neuropathy, *ATTRv-PN* hereditary transthyretin amyloidosis with polyneuropathy, *NA* not available, *NIS* Neuropathy Impairment Score, *NIS-LL* Neuropathy Impairment Score – Lower Limbs, *SD* standard deviation, *SE* standard error

**Table 3:** Summarized Study Characteristics by Disease Type for NIS and NIS-LL Total Scores

Measure	Disease Type	N of Studies	Age^a^: Mean (SD)	Male Gender: %	Initial Assessment Scores^**a**^	
					Mean	SD
NIS						
	CMT	3	41 (18)	45%	60.10	19.80
	DPN	5	56 (8)	53%	13.46	8.70
	ATTRv-PN	4	61 (12)	71%	46.41	38.97
	IPN	1	66 (13)	58%	34.35	22.78
	Small fiber peripheral neuropathy	1	62 (11)	NA	NA	NA
NIS-LL						
	CMT	1	51 (13)	50%	35.88	12.83
	DPN	5	54 (7)	45%	8.83	6.26
	ATTRv-PN	5	48 (11)	61%	15.45	14.56
	IPN	1	66 (13)	58%	24.25	14.06

^a^Means and SDs were calculated across studies for the particular measure and disease type, weighted by individual study sample sizes

*Abbreviations*: *CMT* Charcot-Marie-Tooth disease, *DPN* diabetic peripheral neuropathy, *ATTRv-PN* hereditary transthyretin amyloidosis with polyneuropathy, *NA* not available, *NIS* Neuropathy Impairment Score, *NIS-LL* Neuropathy Impairment Score – Lower Limbs, *SD* standard deviation

### Risk of Bias assessment

**Fig. 2:**
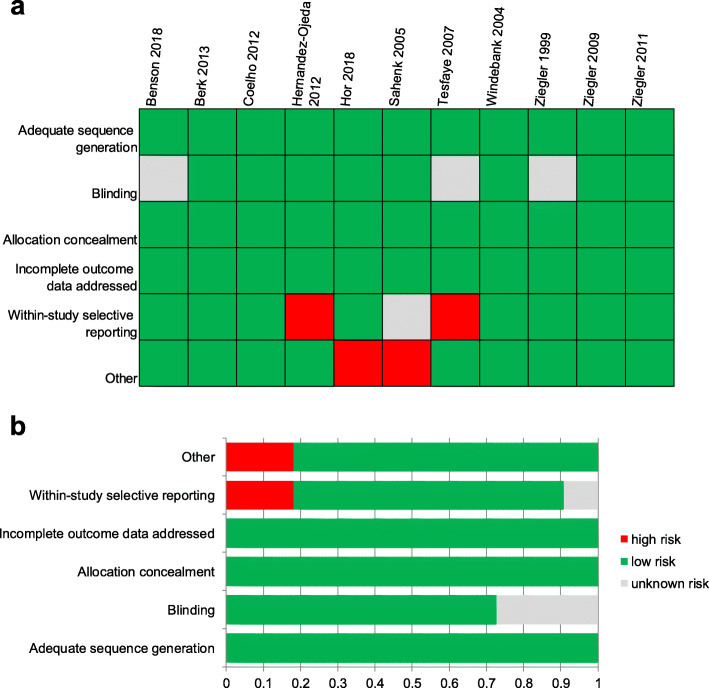
Risk of Bias Assessment for RCTs Included in Data Synthesis: Summary for Items of Bias Within and Across Studies. **a**. Risk of bias for all RCTs included in the meta-analysis presented for individual trials (represented by author name and year) as low, high, or unclear risk of bias in each attribute assessed; **b**. risk of bias for all RCTs included in the meta-analysis presented as the percentages of trials with low, high, or unclear risk of bias for each attribute assessed. Abbreviations: RCT, randomized-controlled trial

The risk of bias for each examined attribute of RCTs included is summarized both within each RCT and across all 11 RCTs in Fig. 2. All RCTs had at least four attributes rated as low risk of bias, and thereby the study-level risk of bias was considered low for all RCTs.

Of the four observational studies, two studies were rated as high risk of bias according to the interpretation of the Newcastle-Ottawa Scale score (scores < 7) [30, 32], with potential sources of bias for these studies related to the selection of groups, the comparability of selected groups, and the determination of outcomes.

### Primary analysis

#### Meta-analysis and meta-regression models for NIS total scores

**Fig. 3:**
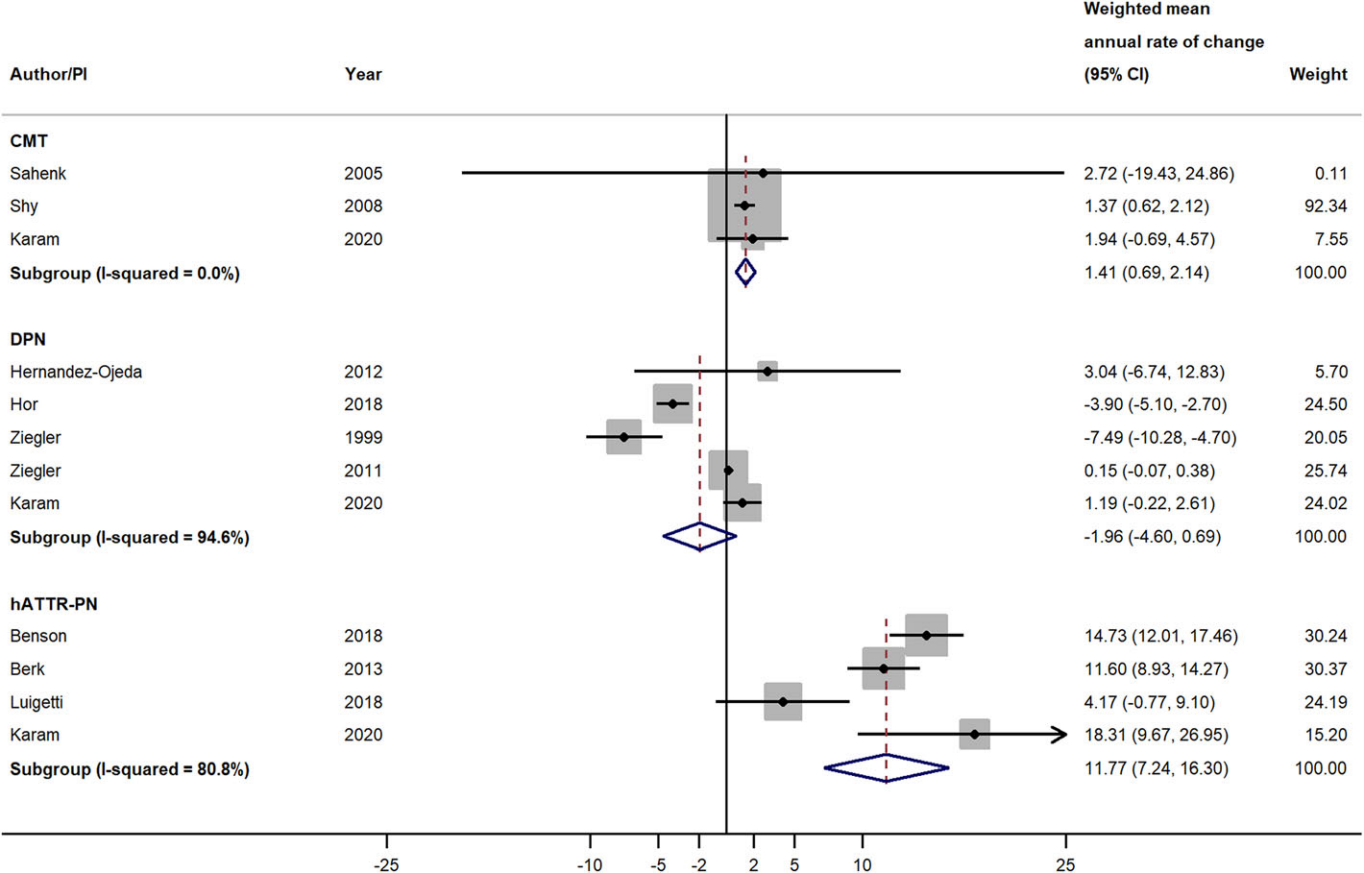
Weighted Mean Annual Rate of Change in NIS Total Score in Studies of Patients with CMT, DPN, and ATTRv-PN. Pooled effect estimates were calculated based on the DerSimonian and Laird random-effects model, weighted by inverse variance. The diamond represents a pooled estimate of the annual rate of change across studies within each disease type. Abbreviations: CI, confidence interval; CMT, Charcot-Marie-Tooth disease; DPN, diabetic peripheral neuropathy; ATTRv-PN, hereditary transthyretin amyloidosis with polyneuropathy; NIS, Neuropathy Impairment Score; PI: principal investigator

Eleven studies reporting changes in NIS total scores were included in the primary analysis. When pooling across studies by disease type, the weighted mean annual rate of change in NIS total score was 11.77 points/year (95% CI = 7.24, 16.30; *P* < 0.001) among studies of patients with ATTRv-PN, − 1.96 points/year (95% CI = − 4.60, 0.69; *P* = 0.147) among studies of patients with DPN, and 1.41 points/year (95% CI = 0.69, 2.14; *P* < 0.001) among studies of patients with CMT (Fig. 3). Meta-analysis was not performed within small fiber peripheral neuropathy or IPN because there was only one study with NIS total scores for each disease. The magnitudes of mean annual rates of change in NIS total scores across studies within each disease type were highly heterogeneous for both ATTRv-PN studies (*I*^2^ = 80.8%) and DPN studies (*I*^2^ = 94.6%), while the CMT studies had an *I*^2^ of 0.0%, which may be due to there being only three studies in this group, with one of those studies having a much larger sample size than the other two, thus dominating the weighting (Fig. 3).

Results from the moderator analyses using meta-regression are summarized in Table 4. The estimated MD of 12.45 points/year (95% CI = 9.34, 15.57; *P* < 0.001) suggests that, on average, studies of patients with ATTRv-PN had a 12.45-point higher annual rate of change in NIS total score as compared to studies of patients with other peripheral neuropathies. After additionally adjusting for NIS total score at initial assessment in the second meta-regression model, the estimated MD was very similar to that in the first model and remained statistically significant (estimated MD = 11.97 points/year, 95% CI = 7.77, 16.18; *P* < 0.001).

**Table 4:** Difference in Estimates of Mean Annual Rate of Change Measured by NIS and NIS-LL between Studies of Patients with ATTRv-PN and Studies of Patients with Other Peripheral Neuropathies

Measure	ATTRv-PN vs. Other peripheral neuropathies^a^			ATTRv-PN vs. Other peripheral neuropathies, adjusting for initial assessment score^**b**^		
	Estimated Difference in Mean Annual Rate of Change	95% CI	***P***	Estimated Difference in Mean Annual Rate of Change	95% CI	***P***
NIS	12.45	9.34, 15.57	< 0.001	11.97	7.77, 16.18	< 0.001
NIS-LL	6.96	4.57, 9.35	< 0.001	6.47	4.01, 8.92	< 0.001

^a^The estimated difference, 95% CI, and *P* value were based on the meta-regression model with the mean annual rate of change in NIS or NIS-LL total score as the dependent variable and the dichotomous variable for disease type (ATTRv-PN vs. other peripheral neuropathies) as the independent variable

^b^The estimated difference, 95% CI, and *P* value were based on the meta-regression model with the mean annual rate of change in NIS or NIS-LL total score as the dependent variable, and the initial assessment score and the dichotomous variable for disease type (ATTRv-PN vs. other peripheral neuropathies) as independent variables

*Abbreviations*: *CI* confidence interval, *ATTRv-PN* hereditary transthyretin amyloidosis with polyneuropathy, *NIS* Neuropathy Impairment Score, *NIS-LL* Neuropathy Impairment Score – Lower Limbs

#### Meta-analysis and meta-regression models for NIS-LL total scores

**Fig. 4:**
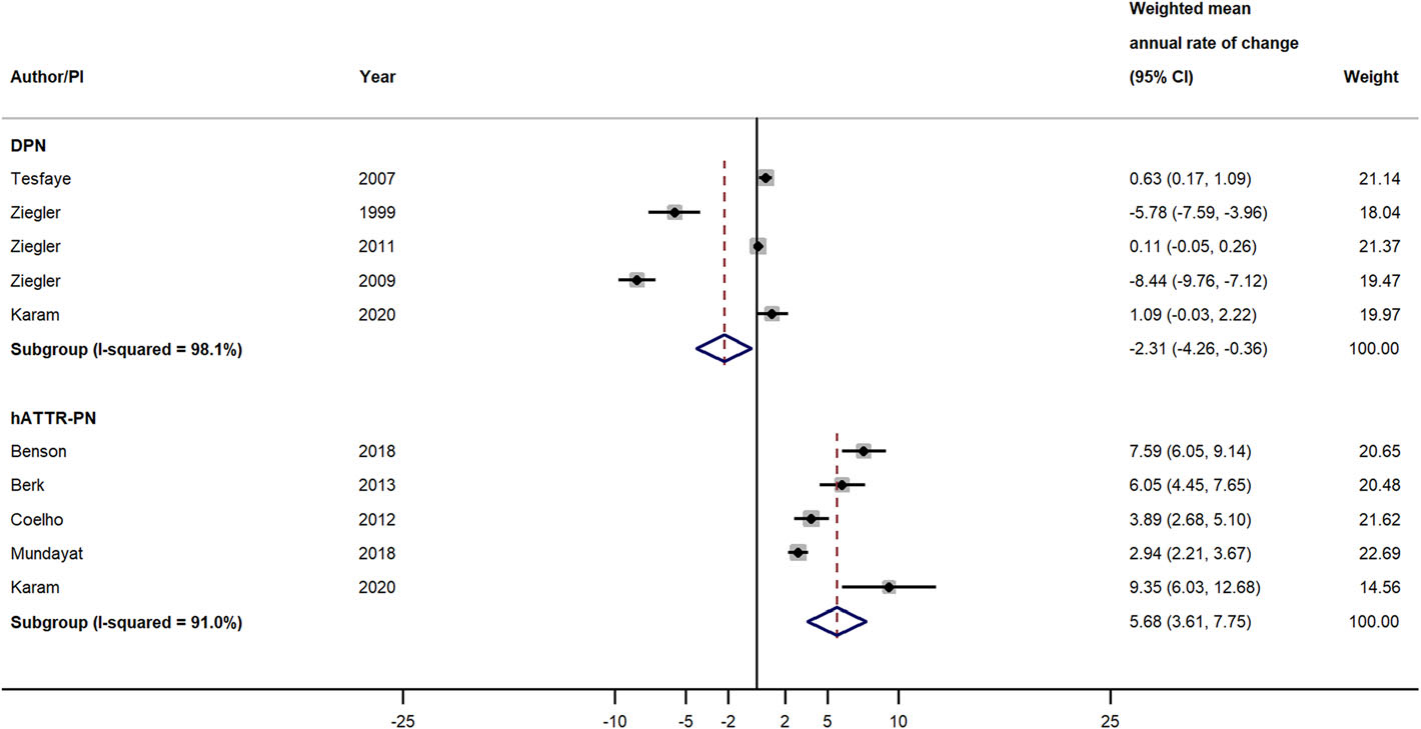
Weighted Mean Annual Rate of Change of NIS-LL Total Score in Studies of Patients with DPN and ATTRv-PN. Pooled effect estimates were calculated based on the DerSimonian and Laird random-effects model, weighted by inverse variance. The diamond represents a pooled estimate of the annual rate of change across studies within disease type. Abbreviations: CI, confidence interval; DPN, diabetic peripheral neuropathy; ATTRv-PN, hereditary transthyretin amyloidosis with polyneuropathy; NIS-LL, Neuropathy Impairment Score – Lower Limbs; PI: principal investigator

Nine studies reporting changes in NIS-LL total scores were included in the primary analysis. When pooling across studies by disease type, the weighted mean annual rate of change in NIS-LL total score was 5.68 points/year (95% CI = 3.61, 7.75; *P* < 0.001) among studies of patients with ATTRv-PN and − 2.31 points/year (95% CI = − 4.26, − 0.36; *P* = 0.020) among studies of patients with DPN (Fig. 4). Meta-analysis was not performed within CMT or IPN because there was only one study with NIS-LL total scores for each disease. The magnitudes of annual rates of change in NIS-LL across studies within each disease type were highly heterogeneous, with *I*^2^ = 91.0% for ATTRv-PN studies and 98.1% for DPN studies (Fig. 4).

Results from the moderator analyses using meta-regression are summarized in Table 4. The estimated MD was 6.96 points/year (95% CI = 4.57, 9.35; *P* < 0.001), suggesting that, on average, the studies of patients with ATTRv-PN had an 6.96-point higher annual rate of change in NIS-LL total score as compared to studies of patients with other peripheral neuropathies. After additionally adjusting for NIS-LL score at initial assessment in the second meta-regression model, the estimated MD was very similar to that in the first model and remained statistically significant (estimated MD = 6.47 points/year, 95% CI = 4.01, 8.92; *P* < 0.001).

### Sensitivity analyses

The weighted mean annual rate of change for each disease type in meta-analysis models for Sensitivity Analyses I, II, and III (Supplementary Table 1) were very similar to those observed in the primary analysis, and the 95% CIs for annual rate of change in NIS and NIS-LL total scores observed in the primary analysis and in all these sensitivity analyses overlapped, suggesting that the potential impacts on findings in the primary analysis of using the last assessment in studies with multiple follow-up assessments, including studies with small sample sizes, and including studies with high risk of bias were not substantial.

Meta-regression models from all sets of sensitivity analyses yielded very similar results in terms of magnitude, direction, and statistical significance of moderator variables as those observed in the primary analysis (Supplementary Table 2).

## Discussion

This systematic review and meta-analysis provide the first, direct empirical comparison of the natural history of neuropathic progression in patients with ATTRv-PN with that of patients with other peripheral neuropathies. Evidence synthesized from 15 studies indicates that patients with ATTRv-PN may experience a more rapid deterioration of peripheral nervous function as compared to patients with other peripheral neuropathies, including DPN and CMT. Specifically, the meta-analysis showed that the annual rate of change in NIS and NIS-LL total scores was significantly different from zero for studies in ATTRv-PN and CMT, but not DPN. Meta-regression, which formally tested the annual rate of change in NIS total scores for studies of patients with ATTRv-PN versus other peripheral neuropathies, indicated significantly faster rate of change in NIS and NIS-LL total scores for patients with ATTRv-PN than patients with other peripheral neuropathies, even when controlling for score at the initial assessment. These findings fit with the known disease course and are consistent with an assessment based on a qualitative review of previous studies using the NIS [12].

In a previous natural history study of 283 patients with ATTRv-PN, investigators predicted a 14.3 points/year increase in NIS total score, as calculated using non-linear models based on the association between the NIS total score and time since diagnosis from a set of cross-sectional data [11]. The predicted annual rate of change reported in that study aligns well with the weighted mean annual rate of change estimated in the current meta-analysis (11.8 points/year) and falls within the corresponding 95% CI for this estimate.

Although the NIS was designed to provide a balance between muscle weakness, reflex loss, and sensory loss, the greatest emphasis is given on muscle weakness [17]. In a previous RCT of patients with ATTRv-PN that reported NIS domain scores, an estimated average change of 17.9 in the NIS-muscle weakness was observed in the placebo arm at 18 months from baseline, showing a dominating role of muscle weakness in the overall neuropathic impairment assessed by a variation of the NIS (an estimated change of 28.0 in the mNIS+ 7 total score in the placebo arm) [9]. Unfortunately, the current research was not able to synthesize evidence for NIS domain scores due to the limited number of studies with available data for each disease type. The lack of the relevant evidence warrants further investigation of whether the observed difference in the rate of neuropathic progression between ATTRv-PN and other peripheral neuropathies was actually driven by a particular domain.

Findings from the current study have several major clinical implications. First, the estimated annual rates of progression could serve as the benchmarks for the clinicians to gauge whether the progressive peripheral neuropathy in a particular patient fits the profile typically seen in ATTRv-PN. In practice, ATTRv-PN is frequently misdiagnosed as CMT, DPN, or IPN in early stages, as these conditions are among the primary causes of distal symmetric peripheral neuropathy [13–16]. The current study indicates that the neuropathic progression in these conditions are much slower than that in ATTRv-PN, and that a progression rate in NIS of 12 or more points per year (i.e., one point per month) is highly suggestive of a typical manifestation of ATTRv-PN. Second, this study gives critical insights on the continuous monitoring of patient response to treatment among patients with ATTRv-PN. The current study, which is focused on patients in placebo arms of RCTs and untreated patients in non-interventional studies, aimed to better understand the natural history of peripheral neuropathy in ATTRv-PN. Hence the annual rates of progression reported in this study well characterized the typical time-course of neuropathic degeneration among patients with untreated ATTRv-PN. Third, because the current study covers the major types of conditions that manifest in peripheral neuropathy with available information in the literature, the evidence and the synthesis will help inform the design of future RCTs for not only ATTRv-PN, but also other peripheral neuropathies.

Aside from this being the first attempt to quantitatively synthesize all available evidence on neuropathic progression in different peripheral neuropathies, the current research has several other strengths. First, meta-regression methodologies were adopted to formally test whether the rate of progression was statistically significantly different between ATTRv-PN and other peripheral neuropathies. Prior to the current research, there has been no such direct empirical comparison, and thus these results provide a concrete interpretive context for the neuropathic degeneration in ATTRv-PN over time. Second, the current research included a comprehensive assessment of robustness with regard to different factors, including the use of the last versus the second-to-last assessment in studies with multiple follow-up assessments (Sensitivity Analysis I), small sample size (Sensitivity Analysis II), and study quality (Sensitivity Analysis III). These sensitivity analyses were intended to evaluate whether and to what extent, potential violations against the assumption of linear changes over time, imprecision introduced by small studies, and risk of bias in individual study design, analysis and reporting, might impact the results from the primary analysis. Results from all sets of sensitivity analyses conducted for the current research were very similar to those from the primary analysis, suggesting that these factors did not impact our findings and conclusions substantially.

This research has some limitations. First, the number of studies available for individual disease type was limited, and we were not able to synthesize evidence for NIS nor NIS-LL domain scores or for other NIS-related measures (e.g., mNIS+ 7) across disease type. Further, the limited number of studies available for synthesis did not afford an assessment of publication bias. Also, ATTRv-PN may have different clinical manifestations depending on various factors, such as TTR genetic mutation (of which more than 140 have been identified worldwide), time since symptom onset, and age of onset (i.e., early or late) [5, 38–41]. This may at least partially account for the substantial heterogeneity observed in the estimated rate of progression across patients and samples. Unfortunately, the small number of studies available for the current research limited our ability to perform any moderator analyses to explore the potential impact of clinically relevant study characteristics as sources of heterogeneity. Second, while we would expect to see increases in NIS and NIS-LL scores over time in untreated patients due to neuropathic progression, the negative estimated annual rate of change in NIS and NIS-LL total scores for studies of patients with DPN (though not significantly different from zero for NIS total scores) suggest a slight improvement over time. One potential explanation for these seemingly discordant findings is that, because all studies synthesized for DPN were actually RCTs, placebo effects might be having positive impacts on patients’ peripheral neuropathy. Supporting this assumption within our data is that the two trials for DPN included in the meta-analysis with the largest decreases in the annual rate of change were the only trials that have between-assessment durations shorter than one year [35, 37]. Extrapolating data from these trials beyond their actual follow-up duration (i.e., calculating an annual rate of change when the follow-up duration is shorter than one year) may amplify the imprecision of the estimate. Third, the potential lack of standardization in the training for performing the NIS-related assessments, especially in the earlier studies of DPN and CMT and in non-interventional studies (as opposed to clinical trials), might lead to biased estimates of the annual rate of progression for those conditions. However, this bias is less likely to impact studies of patients with ATTRv-PN, as most studies of ATTRv-PN were conducted much more recently and as part of clinical trials, for which there is usually more comprehensive training and consistent examiners for these assessments. Unfortunately, details about clinical assessments were not reported in study publications, so our ability to assess the potential bias due to this factor is limited. Fourth, annual rate of change was used so that the current study can synthesize evidence from as many eligible studies as possible with heterogeneous follow-up durations, but the results may potentially be subject to bias by assuming a linear change in the progression of peripheral neuropathy over time. If the underlying linear assumption does not hold, the rate of change might differ substantially across assessments. To explore the potential violation against this assumption, we performed a sensitivity analysis using the second-to-last assessment instead of the last assessment to examine whether the results would differ from the primary findings. Results from this sensitivity analysis were similar to those from the primary analysis in terms of direction and magnitude of the change. Further research, based on longitudinal assessments and using nonlinear models, is needed to evaluate nonlinearity in neuropathic progression over time.

In conclusion, this systematic review and meta-analysis provides empirical evidence suggesting that patients with ATTRv-PN have more rapid neuropathic progression than patients with other peripheral neuropathies, including DPN, CMT, small fiber peripheral neuropathy, and IPN. These findings could help clinicians differentiate ATTRv-PN from other types of peripheral neuropathies, usually with more common and benign etiologies, and to evaluate patient response to treatment in ATTRv-PN. Furthermore, this study may be used to guide the development of new assessment tools and therapies, specifically targeting the rapid progression of peripheral neuropathy in this debilitating disease.

## Supplementary Information

**Additional file 1: Supplementary Table 1**. Weighted Mean Annual Rate of Change in NIS and NIS-LL Total Scores in Studies of Patients with CMT, DPN, and ATTRv-PN – Sensitivity Analyses. **Supplementary Table 2**. Difference in Estimates of Mean Annual Rate of Change in NIS and NIS-LL Total Scores between Studies of Patients with ATTRv-PN and Studies of Patients with Other Peripheral Neuropathies – Sensitivity Analyses.

**Additional file 2.**

## Data Availability

All data generated or analyzed during this study are included in this published article.
