# Supplementary material for: Rate of neuropathic progression in hereditary transthyretin amyloidosis with polyneuropathy and other peripheral neuropathies: a systematic review and meta-analysis
Source: BMC Neurol. 2021 Feb 12;21:70. doi: 10.1186/s12883-021-02094-y (PMC7879641; doi:10.1186/s12883-021-02094-y)
Supplement: Supplementary file 1 — Additional file 1: Supplementary Table 1. Weighted Mean Annual Rate of Change in NIS and NIS-LL Total Scores in Studies of Patients with CMT, DPN, and ATTRv-PN – Sensitivity Analyses. Supplementary Table 2. Difference in Estimates of Mean Annual Rate of Change in NIS and NIS-LL Total Scores between Studies of Patients with ATTRv-PN and Studies of Patients with Other Peripheral Neuropathies – Sensitivity Analyses. [file 12883_2021_2094_MOESM1_ESM.docx]

**Rate of Neuropathic Progression in Hereditary Transthyretin Amyloidosis with Polyneuropathy and Other Peripheral Neuropathies: A Systematic Review and Meta-Analysis**

**Authors:** Xiaochen Lin^1^, Aaron Yarlas^1^, Montserrat Vera-Llonch^2^, Nishtha Baranwal^1^, Josh Biber^1^, Duncan Brown^2^, Braden Vogt^3^, Chafic Karam^3,4^

**Affiliations:** ^1^ QualityMetric, Johnston, RI, USA; ^2^ Akcea Therapeutics, Cambridge, MA, USA; ^3^ Department of Neurology, Oregon Health & Science University, Portland, OR, USA; ^4^ Department of Neurology, University of Pennsylvania, Philadelphia, PA, USA.

**Corresponding author:**

Xiaochen Lin

QualityMetric

1301 Atwood Avenue

Johnston, RI 02919, United States

[xlin@qualitymetric.com](mailto:xlin@qualitymetric.com)

401-642-9202

Supplementary Table 1. Weighted Mean Annual Rate of Change in NIS and NIS-LL Total Scores in Studies of Patients with CMT, DPN, and ATTRv-PN – Sensitivity Analyses

| **Analysis** | | **NIS** | | | |  | **NIS-LL** | | | |  |
| --- | --- | --- | --- | --- | --- | --- | --- | --- | --- | --- | --- |
|  |  | **No. of Studies** | **Weighted Mean Annual Rate of Change^a^** | **95% CI** | ***P*** | ***I^2^*** | **No. of Studies** | **Weighted Mean Annual Rate of Change^a^** | **95% CI** | ***P*** | ***I^2^*** |
| **Sensitivity Analysis I^b^** | |  |  |  |  |  |  |  |  |  |  |
|  | ATTRv-PN | 4 | 10.44 | 6.80, 14.08 | <0.001 | 70.8% | 5 | 5.32 | 3.46, 7.19 | <0.001 | 83.1% |
|  | DPN | 5 | -2.54 | -5.71, 0.63 | 0.117 | 94.0% | 5 | -2.36 | -4.49, -0.22 | 0.03 | 98.1% |
|  | CMT | 3 | 1.41 | 0.69, 2.14 | <0.001 | 0.0% | 1 | - | - | - | - |
| **Sensitivity Analysis II^c^** | |  |  |  |  |  |  |  |  |  |  |
|  | ATTRv-PN | 2 | 13.15 | 10.08, 16.23 | <0.001 | 61.3% | 4 | 5.04 | 2.97, 7.10 | <0.001 | 91.5% |
|  | DPN | 4 | -2.89 | -6.56, 0.77 | 0.122 | 95.8% | 4 | -3.18 | -5.48, -0.88 | 0.007 | 98.5% |
|  | CMT | 1 | - | - | - | - | 0 | - | - | - | - |
| **Sensitivity Analysis III^d^** | |  |  |  |  |  |  |  |  |  |  |
|  | ATTRv-PN | 3 | 13.67 | 10.72, 16.63 | <0.001 | 48.7% | 5 | 5.68 | 3.61, 7.75 | <0.001 | 91.0% |
|  | DPN | 5 | -1.96 | -4.60, 0.69 | 0.147 | 94.6% | 5 | -2.31 | -4.26, -0.36 | 0.020 | 98.1% |
|  | CMT | 2 | 1.95 | -0.66, 4.56 | 0.143 | 0.0% | 1 | - | - | - | - |

^a^Pooled effect estimates, 95% CI, and *P* were based on the DerSimonian and Laird random-effects model, weighted by inverse variance.

^b^Sensitivity Analysis I was performed using the change scores from initial assessment to the second-to-last assessment, instead of the last assessment, from studies with multiple follow-up assessments.

^c^Sensitivity Analysis II was performed by excluding studies with a sample size < 20 [19, 23, 31, 35].

^d^Sensitivity Analysis III was performed by excluding studies rated as high risk of bias [31, 33].

Abbreviations: CI, confidence interval; CMT, Charcot-Marie-Tooth disease; DPN, diabetic peripheral neuropathy; ATTRv-PN, hereditary transthyretin amyloidosis with polyneuropathy; NIS, Neuropathy Impairment Score; NIS-LL, Neuropathy Impairment Score – Lower Limbs.

Supplementary Table 2. Difference in Estimates of Mean Annual Rate of Change in NIS and NIS-LL Total Scores between Studies of Patients with ATTRv-PN and Studies of Patients with Other Peripheral Neuropathies – Sensitivity Analyses

| **Analysis** | | **ATTRv-PN vs. Other peripheral neuropathies^a^** | | | **ATTRv-PN vs. Other peripheral neuropathies, adjusting for initial assessment score^b^** | | |
| --- | --- | --- | --- | --- | --- | --- | --- |
|  |  | **Estimated Difference in Mean Annual Rate of Change** | **95% CI** | ***P*** | **Estimated Difference in Mean Annual Rate of Change** | **95% CI** | ***P*** |
| **Sensitivity Analysis I^c^** | |  |  |  |  |  |  |
|  | NIS | 10.99 | 7.72, 14.25 | <0.001 | 10.61 | 6.07, 15.15 | <0.001 |
|  | NIS-LL | 6.74 | 4.15, 9.33 | <0.001 | 6.39 | 3.68, 9.11 | <0.001 |
| **Sensitivity Analysis II^d^** | |  |  |  |  |  |  |
|  | NIS | 14.98 | 10.94, 19.02 | <0.001 | 13.61 | 6.95, 20.27 | <0.001 |
|  | NIS-LL | 8.23 | 5.02, 11.44 | <0.001 | 6.78 | 2.78, 10.78 | 0.001 |
| **Sensitivity Analysis III^e^** | |  |  |  |  |  |  |
|  | NIS | 14.79 | 10.90, 18.67 | <0.001 | 13.73 | 8.80, 18.65 | <0.001 |
|  | NIS-LL | 6.96 | 4.57, 9.35 | <0.001 | 6.47 | 4.01, 8.92 | <0.001 |

^a^The estimated difference, 95% CI and *P* value were based on the meta-regression model with the mean annual rate of change in NIS or NIS-LL total score as the dependent variable and the dichotomous variable for disease type (ATTRv-PN vs. other peripheral neuropathies) as the independent variable.

^b^The estimated difference, 95% CI and *P* value were based on the meta-regression model with the mean annual rate of change in NIS or NIS-LL total score as the dependent variable, and the initial assessment score and the dichotomous variable for disease type (ATTRv-PN vs. other peripheral neuropathies) as the independent variables.

^c^Sensitivity Analysis I was performed using the change scores from initial assessment to the second-to-last assessment, instead of the last assessment, from studies with multiple follow-up assessments.

^d^Sensitivity Analysis II was performed by excluding studies with a sample size < 20 [19, 23, 31, 35].

^e^Sensitivity Analysis III was performed by excluding studies rated as high risk of bias [31, 33].

Abbreviations: CI, confidence interval; ATTRv-PN, hereditary transthyretin amyloidosis with polyneuropathy; NIS: Neuropathy Impairment Score; NIS-LL, Neuropathy Impairment Score – Lower Limbs.
