# Supplementary material for: Rate of neuropathic progression in hereditary transthyretin amyloidosis with polyneuropathy and other peripheral neuropathies: a systematic review and meta-analysis
Source: BMC Neurol. 2021 Feb 12;21:70. doi: 10.1186/s12883-021-02094-y (PMC7879641; doi:10.1186/s12883-021-02094-y)
Supplement: Supplementary file 2 — Additional file 2. [file 12883_2021_2094_MOESM2_ESM.docx]

**Rate of Neuropathic Progression in Hereditary Transthyretin Amyloidosis with Polyneuropathy and Other Peripheral Neuropathies: A Systematic Review and Meta-Analysis**

**Authors:** Xiaochen Lin^1^, Aaron Yarlas^1^, Montserrat Vera-Llonch^2^, Nishtha Baranwal^1^, Josh Biber^1^, Duncan Brown^2^, Braden Vogt^3^, Chafic Karam^3,4^

**Affiliations:** ^1^ QualityMetric, Johnston, RI, USA; ^2^ Akcea Therapeutics, Cambridge, MA, USA; ^3^ Department of Neurology, Oregon Health & Science University, Portland, OR, USA; ^4^ Department of Neurology, University of Pennsylvania, Philadelphia, PA, USA.

**Corresponding author:**

Xiaochen Lin

QualityMetric

1301 Atwood Avenue

Johnston, RI 02919, United States

[xlin@qualitymetric.com](mailto:xlin@qualitymetric.com)

401-642-9202

# Searches Conducted for Standardized Literature Review Commissioned by Akcea Therapeutics

## Electronic Searches

The electronic searches were performed in PubMed, EmBase and the Cochrane library to identify all relevant publications related to patients with ATTRv reporting data on epidemiology, natural history, humanistic burden, economic burden, and management, together with clinical evidence for inotersen, patisiran, tafamidis, diflunisal, and liver transplantation (LT) as treatment for ATTRv. Searches were restricted to publications regarding humans in English language journals from 2008 to 2018.

Table 1: Databases included in the preliminary search for SRs

| **Database** | **Platform** | **Span of search** | **Date searched** |
| --- | --- | --- | --- |
| EmBase | Embase.com | 2008-2018 | 7-Feburary-2018 |
| PubMed | [www.ncbi.nlm.nih.gov/pubmed/](http://www.ncbi.nlm.nih.gov/pubmed/) | 2008-2018 | 31-January-2018 |
| Cochrane library | <http://onlinelibrary.wiley.com/cochranelibrary/search/> | 2008-2018 | 2-Feburary-2018 |

The following tables describe the suggested search strings and the number of hits. Similar searches were also conducted in EmBase, Cochrane and other databases to obtain the final list of the citations to be screened.

**PubMed search strings and the number of hits (31 Jan 2018)**

Estimated number to be screened: 4122 (#1)

Estimated number for ATTRv-PN with any outcome: 1246 (#27)

Estimated number for ATTRv-PN with any intervention: 229 (#11)

| **Search** | **Query** | **Items found** |
| --- | --- | --- |
| #27 | #1 AND #26 | 1246 |
| #26 | #19 OR #21 OR #23 OR #25 | 7097719 |
| #25 | Incidenc*[tiab] OR "Prevalence"[tiab] OR epidem*[tiab] OR mortalit*[tiab] OR natural histor*[tiab] OR demograph*[tiab] OR morbid*[tiab] OR risk[tiab] OR survival[tiab] OR etiology[tiab] OR aetiology[tiab] OR distribution[tiab] OR Frequency[tiab] OR pattern[tiab] | 5572811 |
| #24 | #11 AND #23 | 2 |
| #23 | "resource use"[tiab] OR resource utili*[tiab] OR "resource usage"[tiab] OR "nursing cost"[tiab] OR "resource allocation"[tiab] OR "resource management"[tiab] OR "Health Care"[tiab] OR Health Care cost*[tiab] OR "direct cost"[tiab] OR "indirect cost"[tiab] OR "Economic burden"[tiab] OR "economic impact"[tiab] OR "Disease Burden"[tiab] OR "Burden of illness"[tiab] OR "Burden of sickness"[tiab] OR "Sickness Burden"[tiab] OR "burden of disease"[tiab] OR productivit*[tiab] OR "productivity"[tiab] OR "work day"[tiab] OR "working days"[tiab] OR "length of stay"[tiab] OR "duration of stay"[tiab] OR "extended stay"[tiab] OR "prolonged stay"[tiab] OR "duration of stay"[tiab] OR "prolonged stay"[tiab] OR "duration of hospitalisation"[tiab] OR "bed-days"[tiab] OR "bed days"[tiab] OR re-admi*[tiab] OR readmi*[tiab] OR "readmission"[tiab] OR "hospital readmission"[tiab] OR "ICU stay"[tiab] OR "ICU day"[tiab] OR absent*[tiab] OR "absenteeism"[tiab] OR "presenteeism"[tiab] OR "work day"[tiab] OR "working days"[tiab] OR "Lost Work productivity"[tiab] | 614300 |
| #22 | #11 AND #21 | 25 |
| #21 | "Quality of life" OR "HRQOL" OR "QOL" OR "HRQL" OR "health related quality of life" OR "health utilities index" OR "HUI" | 284766 |
| #20 | #11 AND #19 | 6 |
| #19 | #12 OR #15 OR #16 OR #17 OR #18 | 1544027 |
| #18 | Economics[Mesh] OR Economic*[Mesh] OR Economic*[tiab] OR Cost[Mesh] OR cost[tiab] OR "costs and cost analysis"[Mesh] OR "costs and cost analysis"[tiab] OR "Cost allocation"[tiab] OR "Cost-allocation"[tiab] OR "Cost-benefit analysis"[tiab] OR "Cost control"[tiab] OR "Cost savings"[tiab] OR "Cost of illness"[tiab] OR "Cost sharing"[tiab] OR "deductibles and coinsurance"[tiab] OR "Medical savings accounts"[tiab] OR "Health care costs"[tiab] OR "Direct service costs"[tiab] OR "Drug costs"[tiab] OR "Employer health costs"[tiab] OR "Hospital costs"[tiab] OR "Health expenditures"[tiab] OR "Capital expenditures"[tiab] OR "Value of life"[tiab] OR "fees and charges"[tiab] OR charg*[tiab] OR fees[tiab] OR budget[Mesh] OR budget[tiab] OR "fiscal"[tiab] OR fund*[tiab] OR financ*[tiab] OR "cost estimate"[tiab] OR "cost-estimate"[tiab] OR "cost variable"[tiab] OR "cost-variable"[tiab] OR "variable cost"[tiab] OR "variable-cost"[tiab] OR "unit cost"[tiab] OR "unit-cost"[tiab] OR pharmacoeconomic[tiab] OR "pharmaco economic"[tiab] OR "pharmaco-economic"[tiab] OR pric*[tiab] OR cost- effectiv*[tiab] OR cost effectiv*[tiab] OR cost-effectiv*[tiab] OR "cost effectiveness"[tiab] OR "cost-effectiveness"[tiab] OR "Socioeconomic"[tiab] OR "Cost utility"[tiab] OR "cost minimization"[tiab] OR "cost-minimization"[tiab] OR "health care utilisation"[tiab] OR "economic aspect"[tiab] OR "financial management"[tiab] OR "health economics"[tiab] OR reimburse*[tiab] OR re-imburse*[tiab] | 1523372 |
| #17 | "fiscal"[tiab] OR "funding"[tiab] OR "financial"[tiab] OR "finance"[tiab] OR economic*[tiab] OR pharmacoeconomi*[tiab] OR price*[tiab] OR "pricing"[tiab] | 365001 |
| #16 | "Low cost"[tiab] OR "High cost"[tiab] OR "Low costs"[tiab] OR "High costs"[tiab] OR "Health care cost"[tiab] OR "healthcare cost"[tiab] OR "health-care cost"[tiab] OR "Health care costs"[tiab] OR "healthcare costs"[tiab] OR "health-care costs"[tiab] OR "Estimated cost"[tiab] OR "Estimated costs"[Tiab] OR cost-Estimat*[tiab] OR "Cost-Estimate"[tiab] OR "Cost-Estimates"[tiab] OR "Variable cost"[tiab] OR "Unit cost"[tiab] OR "Variable costs"[tiab] OR "Unit costs"[tiab] OR "cost-Estimation"[tiab] OR "Cost per unit"[Tiab] | 82953 |
| #15 | #13 AND #14 | 154062 |
| #14 | Economic*[tiab] OR Cost*[tiab] | 675306 |
| #13 | "hospital" [tiab] OR "medical" [tiab] OR "nursing" [tiab] OR "pharmaceutical" [tiab] | 1958642 |
| #12 | Economic*[tiab] OR "Economic"[MeSH] OR "Economics"[tiab] OR "Economics"[MeSH] OR "costs and cost analysis"[tiab] OR "Cost allocation"[tiab] OR "Cost-benefit analysis"[tiab] OR "Cost control"[tiab] OR "Cost savings"[tiab] OR "Cost of illness"[tiab] OR "Cost sharing"[tiab] OR "deductibles and coinsurance"[tiab] OR "deductibles"[tiab] OR "coinsurance"[tiab] OR "Medical savings accounts"[tiab] OR "Health care costs"[tiab] OR "Direct service costs"[tiab] OR "Drug costs"[tiab] OR "Employer health costs"[tiab] OR "Hospital costs"[tiab] OR "Health expenditures"[tiab] OR "Capital expenditure"[tiab] OR "Value of life"[tiab] OR "fees and charges"[tiab] OR "budget"[tiab] | 741930 |
| **#11** | **#1 AND #10** | **229** |
| #10 | #2 OR #3 OR #4 OR #5 OR #6 OR #7 OR #8 OR #9 | 32666 |
| #9 | "Liver transplant" OR "hepatic transplant" | 16375 |
| #8 | "Doxycycline/tauroursodeoxycholic acid" OR "Doxy-TUDCA" OR "Tauroursodeoxycholic Acid and Doxycycline" OR "Doxycycline and tauroursodeoxycholic Acid" OR "Doxycycline and tau-URSO" OR "Doxycycline + tau-URSO" OR "Doxycycline + tauroursodeoxycholic acid" | 14864 |
| #7 | "revusiran" OR "Revusiran [INN]" | 4 |
| #6 | "IONIS-TTRRx" OR "ISIS 420915" OR "IONIS-TTR" OR Inotersen OR "GSK2998728" OR "UNII-950736UC77" OR "IONISTTRRx" OR "ISIS420915" OR "IONISTTR" OR "GSK-2998728" OR "UNII950736UC77" OR "IONIS TTRRx" OR "ISIS-420915" OR "IONIS TTR" OR "UNII 950736UC77" | 185 |
| #5 | "tolcapone" OR "SOM-0226" OR "SOM0226" | 424 |
| #4 | "patisiran" OR "ALN-TTR02" OR "ALN-18328" OR "UNII-50FKX8CB2Y" OR "ALNTTR02" OR "ALN18328" OR "UNII50FKX8CB2Y" OR "ALN TTR02" OR "ALN 18328" OR "UNII 50FKX8CB2Y" | 194 |
| #3 | "diflunisal" OR "Dolobid" OR "Algobid" OR "MK 647" OR "MK-647" OR "Diflunisalum" OR "MK647" | 750 |
| #2 | "Tafamidis" OR "2-(3,5-dichloro-phenyl)-benzoxazole-6-carboxylic acid" OR "Vyndaqel" OR "Fx-1006" | 106 |
| #1 | "Familial Amyloid Polyneuropathy" OR "hereditary TTR amyloid polyneuropathy" OR "hATTR-Polyneuropathy" OR "hATTR-PN" OR "hATTR Polyneuropathy" OR "hATTR PN" OR "Transthyretin amyloidosis" OR "transthyretin-related hereditary amyloidosis" OR "TTR-FAP" OR "transthyretin familial polyneuropathy" OR "Transthyretin amyloid neuropathy" OR "Amyloidosis Transthyretin related" OR "Neuropathic heredofamilial amyloidosis" OR "Neuropathic heredofamilial amyloidosis" OR "Transthyretin amyloid polyneuropathy" OR "TTR amyloid neuropathy" OR "Familial transthyretin amyloidosis" OR "Familial amyloid neuropathies" OR “Amyloid Polyneuropathy” OR “Amyloid Polyneuropathies” OR “transthyretin polyneuropathy” OR “transthyretin polyneuropathies” OR "Familial polyneuropathies" OR “Familial polyneuropathy" OR “Amyloid neuropathy” OR “Amyloid neuropathies” OR “transthyretin neuropathy” OR “transthyretin neuropathies” OR "Familial neuropathies" OR “Familial neuropathy" OR “Amyloid cardiomyopathy” OR “Amyloid cardiomyopathies” OR “transthyretin cardiomyopathy” OR "transthyretin cardiomyopathies” OR "Familial cardiomyopathies" OR “Familial cardiomyopathy” OR "hATTR cardiomyopathy" OR "hATTR cardiomyopathies" OR "hATTR neuropathy" OR "hATTR neuropathies"OR "hATTR polyneuropathy" OR "hATTR polyneuropathies" OR “Familial Amyloid Cardiomyopathy” OR “Cardiac amyloidosis” | 4122 |

**Embase search strings and the number of hits (7 Feb 2018)**

| **Search** | **Query** | **Items found** |
| --- | --- | --- |
| #29 | ('familial amyloid polyneuropathy' OR 'hereditary ttr amyloid polyneuropathy' OR 'hattr-polyneuropathy' OR 'hattr-pn' OR 'hattr pn' OR 'transthyretin amyloidosis' OR 'transthyretin-related hereditary amyloidosis' OR 'ttr-fap' OR 'transthyretin familial polyneuropathy' OR 'transthyretin amyloid neuropathy' OR 'amyloidosis transthyretin related' OR 'neuropathic heredofamilial amyloidosis' OR 'transthyretin amyloid polyneuropathy' OR 'ttr amyloid neuropathy' OR 'familial transthyretin amyloidosis' OR 'familial amyloid neuropathies' OR 'amyloid polyneuropathy' OR 'amyloid polyneuropathies' OR 'transthyretin polyneuropathy' OR 'transthyretin polyneuropathies' OR 'familial polyneuropathies' OR 'familial polyneuropathy' OR 'amyloid neuropathy' OR 'amyloid neuropathies' OR 'transthyretin neuropathy' OR 'transthyretin neuropathies' OR 'familial neuropathies' OR 'familial neuropathy' OR 'amyloid cardiomyopathy' OR 'amyloid cardiomyopathies' OR 'transthyretin cardiomyopathy' OR 'transthyretin cardiomyopathies' OR 'familial cardiomyopathies' OR 'familial cardiomyopathy' OR 'hattr cardiomyopathy' OR 'hattr cardiomyopathies' OR 'hattr neuropathy' OR 'hattr neuropathies' OR 'hattr polyneuropathy' OR 'hattr polyneuropathies' OR 'familial amyloid cardiomyopathy' OR 'cardiac amyloidosis') AND ([young adult]/lim OR [adult]/lim OR [middle aged]/lim OR [aged]/lim OR [very elderly]/lim) AND [humans]/lim AND [2008-2018]/py | 1513 |
| #28 | #1 AND #27 | 2152 |
| #27 | #20 OR #22 OR #24 OR #26 | 7571489 |
| #26 | (incidenc*:ab,ti OR 'prevalence':ab,ti OR epidem*:ab,ti OR mortalit*:ab,ti OR natural) AND histor*:ab,ti OR demograph*:ab,ti OR morbid*:ab,ti OR risk:ab,ti OR survival:ab,ti OR etiology:ab,ti OR aetiology:ab,ti OR distribution:ab,ti OR frequency:ab,ti OR pattern:ab,ti | 6111335 |
| #25 | #12 AND #24 | 26 |
| #24 | (('resource use':ab,ti OR resource) AND utili*:ab,ti OR 'resource usage':ab,ti OR 'nursing cost':ab,ti OR 'resource allocation':ab,ti OR 'resource management':ab,ti OR 'health care':ab,ti OR health) AND care AND cost*:ab,ti OR 'direct cost':ab,ti OR 'indirect cost':ab,ti OR 'economic burden':ab,ti OR 'economic impact':ab,ti OR 'disease burden':ab,ti OR 'burden of illness':ab,ti OR 'burden of sickness':ab,ti OR 'sickness burden':ab,ti OR 'burden of disease':ab,ti OR productivit*:ab,ti OR 'productivity':ab,ti OR 'length of stay':ab,ti OR 'extended stay':ab,ti OR 'duration of stay':ab,ti OR 'prolonged stay':ab,ti OR 'duration of hospitalisation':ab,ti OR 'bed-days':ab,ti OR 'bed days':ab,ti OR 're admi*':ab,ti OR readmi*:ab,ti OR 'readmission':ab,ti OR 'hospital readmission':ab,ti OR 'icu stay':ab,ti OR 'icu day':ab,ti OR absent*:ab,ti OR 'absenteeism':ab,ti OR 'presenteeism':ab,ti OR 'work day':ab,ti OR 'working days':ab,ti OR 'lost work productivity':ab,ti | 596060 |
| #23 | #12 AND #22 | 95 |
| #22 | 'quality of life' OR 'hrqol' OR 'qol' OR 'hrql' OR 'health related quality of life' OR 'health utilities index' OR 'hui' | 641286 |
| #21 | #12 AND #20 | 15 |
| #20 | #13 OR #16 OR #17 OR #18 OR #19 | 1168997 |
| #19 | (('economics'/exp OR economic*:ab,ti OR 'cost'/exp OR cost:ab,ti OR 'costs and cost analysis'/exp OR 'costs and cost analysis':ab,ti OR 'cost allocation':ab,ti OR 'cost-allocation':ab,ti OR 'cost-benefit analysis':ab,ti OR 'cost control':ab,ti OR 'cost savings':ab,ti OR 'cost of illness':ab,ti OR 'cost sharing':ab,ti OR 'deductibles and coinsurance':ab,ti OR 'medical savings accounts':ab,ti OR 'health care costs':ab,ti OR 'direct service costs':ab,ti OR 'drug costs':ab,ti OR 'employer health costs':ab,ti OR 'hospital costs':ab,ti OR 'health expenditures':ab,ti OR 'capital expenditures':ab,ti OR 'value of life':ab,ti OR 'fees and charges':ab,ti OR charg*:ab,ti OR fees:ab,ti OR 'budget'/exp OR budget:ab,ti OR 'fiscal':ab,ti OR fund*:ab,ti OR financ*:ab,ti OR 'cost estimate':ab,ti OR 'cost-estimate':ab,ti OR 'cost variable':ab,ti OR 'cost-variable':ab,ti OR 'variable cost':ab,ti OR 'variable-cost':ab,ti OR 'unit cost':ab,ti OR 'unit-cost':ab,ti OR pharmacoeconomic:ab,ti OR 'pharmaco economic':ab,ti OR 'pharmaco-economic':ab,ti OR pric*:ab,ti OR cost-) AND effectiv*:ab,ti OR cost) AND effectiv*:ab,ti OR 'cost effectiv*':ab,ti OR 'cost effectiveness':ab,ti OR 'cost-effectiveness':ab,ti OR 'socioeconomic':ab,ti OR 'cost utility':ab,ti OR 'cost minimization':ab,ti OR 'cost-minimization':ab,ti OR 'health care utilisation':ab,ti OR 'economic aspect':ab,ti OR 'financial management':ab,ti OR 'health economics':ab,ti OR reimburse*:ab,ti OR 're imburse*':ab,ti | 419486 |
| #18 | 'fiscal':ab,ti OR 'funding':ab,ti OR 'financial':ab,ti OR 'finance':ab,ti OR economic*:ab,ti OR pharmacoeconomi*:ab,ti OR price*:ab,ti OR 'pricing':ab,ti | 448615 |
| #17 | 'low cost':ab,ti OR 'high cost':ab,ti OR 'low costs':ab,ti OR 'high costs':ab,ti OR 'health care cost':ab,ti OR 'healthcare cost':ab,ti OR 'health-care cost':ab,ti OR 'health care costs':ab,ti OR 'healthcare costs':ab,ti OR 'health-care costs':ab,ti OR 'estimated cost':ab,ti OR 'estimated costs':ab,ti OR 'cost estimat*':ab,ti OR 'cost-estimate':ab,ti OR 'cost-estimates':ab,ti OR 'variable cost':ab,ti OR 'unit cost':ab,ti OR 'variable costs':ab,ti OR 'unit costs':ab,ti OR 'cost-estimation':ab,ti OR 'cost per unit':ab,ti | 105709 |
| #16 | #14 AND #15 | 227566 |
| #15 | economic*:ab,ti OR cost*:ab,ti | 851444 |
| #14 | 'hospital':ab,ti OR 'medical':ab,ti OR 'nursing':ab,ti OR 'pharmaceutical':ab,ti | 2755068 |
| #13 | economic OR 'economic' OR 'economics':ab,ti OR 'economics'/exp OR 'costs and cost analysis':ab,ti OR 'cost allocation':ab,ti OR 'cost-benefit analysis':ab,ti OR 'cost control':ab,ti OR 'cost savings':ab,ti OR 'cost of illness':ab,ti OR 'cost sharing':ab,ti OR 'deductibles and coinsurance':ab,ti OR 'deductibles':ab,ti OR 'coinsurance':ab,ti OR 'medical savings accounts':ab,ti OR 'health care costs':ab,ti OR 'direct service costs':ab,ti OR 'drug costs':ab,ti OR 'employer health costs':ab,ti OR 'hospital costs':ab,ti OR 'health expenditures':ab,ti OR 'capital expenditure':ab,ti OR 'value of life':ab,ti OR 'fees and charges':ab,ti OR 'budget':ab,ti | 602711 |
| #12 | #1 AND #11 | 646 |
| #11 | #3 OR #4 OR #5 OR #6 OR #7 OR #8 OR #9 OR #10 | 41795 |
| #10 | 'liver transplant' OR 'hepatic transplant' | 37153 |
| #9 | 'doxycycline/tauroursodeoxycholic acid' OR 'doxy-tudca' OR 'tauroursodeoxycholic acid and doxycycline' OR 'doxycycline and tauroursodeoxycholic acid' OR 'doxycycline and tau-urso' OR 'doxycycline + tau-urso' OR 'doxycycline + tauroursodeoxycholic acid' | 7 |
| #8 | 'revusiran' OR 'revusiran [inn]' | 23 |
| #7 | 'ionis-ttrrx' OR 'isis 420915' OR 'ionis-ttr' OR inotersen OR 'gsk2998728' OR 'unii-950736uc77' OR 'ionisttrrx' OR 'isis420915' OR 'ionisttr' OR 'gsk-2998728' OR 'unii950736uc77' OR 'ionis ttrrx' OR 'isis-420915' OR 'ionis ttr' OR 'unii 950736uc77' | 20 |
| #6 | 'tolcapone' OR 'som-0226' OR 'som0226' | 1684 |
| #5 | 'patisiran' OR 'aln-ttr02' OR 'aln-18328' OR 'unii-50fkx8cb2y' OR 'alnttr02' OR 'aln18328' OR 'unii50fkx8cb2y' OR 'aln ttr02' OR 'aln 18328' OR 'unii 50fkx8cb2y' | 78 |
| #4 | 'diflunisal' OR 'dolobid' OR 'algobid' OR 'mk 647' OR 'mk-647' OR 'diflunisalum' OR 'mk647' | 2670 |
| #3 | 'tafamidis' OR '2-(3,5-dichloro-phenyl)-benzoxazole-6-carboxylic acid' OR 'vyndaqel' OR 'fx-1006' | 373 |
| #2 | ('familial amyloid polyneuropathy' OR 'hereditary ttr amyloid polyneuropathy' OR 'hattr-polyneuropathy' OR 'hattr-pn' OR 'hattr pn' OR 'transthyretin amyloidosis' OR 'transthyretin-related hereditary amyloidosis' OR 'ttr-fap' OR 'transthyretin familial polyneuropathy' OR 'transthyretin amyloid neuropathy' OR 'amyloidosis transthyretin related' OR 'neuropathic heredofamilial amyloidosis' OR 'transthyretin amyloid polyneuropathy' OR 'ttr amyloid neuropathy' OR 'familial transthyretin amyloidosis' OR 'familial amyloid neuropathies' OR 'amyloid polyneuropathy' OR 'amyloid polyneuropathies' OR 'transthyretin polyneuropathy' OR 'transthyretin polyneuropathies' OR 'familial polyneuropathies' OR 'familial polyneuropathy' OR 'amyloid neuropathy' OR 'amyloid neuropathies' OR 'transthyretin neuropathy' OR 'transthyretin neuropathies' OR 'familial neuropathies' OR 'familial neuropathy' OR 'amyloid cardiomyopathy' OR 'amyloid cardiomyopathies' OR 'transthyretin cardiomyopathy' OR 'transthyretin cardiomyopathies' OR 'familial cardiomyopathies' OR 'familial cardiomyopathy' OR 'hattr cardiomyopathy' OR 'hattr cardiomyopathies' OR 'hattr neuropathy' OR 'hattr neuropathies' OR 'hattr polyneuropathy' OR 'hattr polyneuropathies' OR 'familial amyloid cardiomyopathy' OR 'cardiac amyloidosis') AND ([young adult]/lim OR [adult]/lim OR [middle aged]/lim OR [aged]/lim OR [very elderly]/lim) AND [humans]/lim | 2706 |
| #1 | 'familial amyloid polyneuropathy' OR 'hereditary ttr amyloid polyneuropathy' OR 'hattr-polyneuropathy' OR 'hattr-pn' OR 'hattr pn' OR 'transthyretin amyloidosis' OR 'transthyretin-related hereditary amyloidosis' OR 'ttr-fap' OR 'transthyretin familial polyneuropathy' OR 'transthyretin amyloid neuropathy' OR 'amyloidosis transthyretin related' OR 'neuropathic heredofamilial amyloidosis' OR 'transthyretin amyloid polyneuropathy' OR 'ttr amyloid neuropathy' OR 'familial transthyretin amyloidosis' OR 'familial amyloid neuropathies' OR 'amyloid polyneuropathy' OR 'amyloid polyneuropathies' OR 'transthyretin polyneuropathy' OR 'transthyretin polyneuropathies' OR 'familial polyneuropathies' OR 'familial polyneuropathy' OR 'amyloid neuropathy' OR 'amyloid neuropathies' OR 'transthyretin neuropathy' OR 'transthyretin neuropathies' OR 'familial neuropathies' OR 'familial neuropathy' OR 'amyloid cardiomyopathy' OR 'amyloid cardiomyopathies' OR 'transthyretin cardiomyopathy' OR 'transthyretin cardiomyopathies' OR 'familial cardiomyopathies' OR 'familial cardiomyopathy' OR 'hattr cardiomyopathy' OR 'hattr cardiomyopathies' OR 'hattr neuropathy' OR 'hattr neuropathies' OR 'hattr polyneuropathy' OR 'hattr polyneuropathies' OR 'familial amyloid cardiomyopathy' OR 'cardiac amyloidosis' | 6286 |

**Cochrane search strings and the number of hits (2 Feb 2018)**

| **Search** | **Query** | **Items found** |
| --- | --- | --- |
| #1 | Familial Amyloid Polyneuropathy or "hereditary TTR amyloid polyneuropathy" or "hATTR-Polyneuropathy" or "hATTR-PN" or "hATTR Polyneuropathy" or "hATTR PN" or "Transthyretin amyloidosis" or "transthyretin-related hereditary amyloidosis" or "TTR-FAP" or "transthyretin familial polyneuropathy" or "Transthyretin amyloid neuropathy" or "Amyloidosis Transthyretin related" or "Neuropathic heredofamilial amyloidosis" or "Neuropathic heredofamilial amyloidosis" or "Transthyretin amyloid polyneuropathy" or "TTR amyloid neuropathy" or "Familial transthyretin amyloidosis" or "Familial amyloid neuropathies" or "Amyloid Polyneuropathy" or "Amyloid Polyneuropathies" or "transthyretin polyneuropathy" or "transthyretin polyneuropathies" or "Familial polyneuropathies" or "Familial polyneuropathy" or "Amyloid neuropathy" or "Amyloid neuropathies" or "transthyretin neuropathy" or "transthyretin neuropathies" or "Familial neuropathies" or "Familial neuropathy" or "Amyloid cardiomyopathy" or "Amyloid cardiomyopathies" or "transthyretin cardiomyopathy" or "transthyretin cardiomyopathies" or "Familial cardiomyopathies" or "Familial cardiomyopathy" or "hATTR cardiomyopathy" or "hATTR cardiomyopathies" or "hATTR neuropathy" or "hATTR neuropathies" or "hATTR polyneuropathy" or "hATTR polyneuropathies" or "Familial Amyloid Cardiomyopathy" or "Cardiac amyloidosis" (Word variations have been searched) | 109 |
| #2 | Tafamidis or "2-(3,5-dichloro-phenyl)-benzoxazole-6-carboxylic acid" or "Vyndaqel" or "Fx-1006" | 46 |
| #3 | diflunisal or "Dolobid" or "Algobid" or "MK 647" or "MK-647" or "Diflunisalum" or "MK647" | 301 |
| #4 | patisiran or "ALN-TTR02" or "ALN-18328" or "UNII-50FKX8CB2Y" or "ALNTTR02" or "ALN18328" or "UNII50FKX8CB2Y" or "ALN TTR02" or "ALN 18328" or "UNII 50FKX8CB2Y" | 15 |
| #5 | tolcapone or "SOM-0226" or "SOM0226" | 112 |
| #6 | IONIS-TTRRx or "ISIS 420915" or "IONIS-TTR" or Inotersen or "GSK2998728" or "UNII-950736UC77" or "IONISTTRRx" or "ISIS420915" or "IONISTTR" or "GSK-2998728" or "UNII950736UC77" or "IONIS TTRRx" or "ISIS-420915" or "IONIS TTR" or "UNII 950736UC77" | 4 |
| #7 | revusiran or "Revusiran [INN]" | 2 |
| #8 | Doxycycline/tauroursodeoxycholic acid or "Doxy-TUDCA" or "Tauroursodeoxycholic Acid and Doxycycline" or "Doxycycline and tauroursodeoxycholic Acid" or "Doxycycline and tau-URSO" or "Doxycycline + tau-URSO" or "Doxycycline + tauroursodeoxycholic acid" | 0 |
| #9 | Liver transplant or "hepatic transplant" | 1368 |
| #10 | #2 or #3 or #4 or #5 or #6 or #7 or #8 or #9 | 1824 |
| #11 | #1 and #10 | 60 |
| #12 | Economic* or "Economic" or "Economics" or "Economics" or "costs and cost analysis" or "Cost allocation" or "Cost-benefit analysis" or "Cost control" or "Cost savings" or "Cost of illness" or "Cost sharing" or "deductibles and coinsurance" or "deductibles" or "coinsurance" or "Medical savings accounts" or "Health care costs" or "Direct service costs" or "Drug costs" or "Employer health costs" or "Hospital costs" or "Health expenditures" or "Capital expenditure" or "Value of life" or "fees and charges" or "budget":ti,ab,kw (Word variations have been searched) | 41523 |
| #13 | hospital or "medical" or "nursing" or "pharmaceutical":ti,ab,kw (Word variations have been searched) | 182341 |
| #14 | Economic* or Cost* | 88696 |
| #15 | #13 and #14 | 26467 |
| #16 | Low cost or "High cost" or "Low costs" or "High costs" or "Health care cost" or "healthcare cost" or "health-care cost" or "Health care costs" or "healthcare costs" or "health-care costs" or "Estimated cost" or "Estimated costs" or cost-Estimat* or "Cost-Estimate" or "Cost-Estimates" or "Variable cost" or "Unit cost" or "Variable costs" or "Unit costs" or "cost-Estimation" or "Cost per unit":ti,ab,kw (Word variations have been searched) | 13710 |
| #17 | fiscal or "funding" or "financial" or "finance" or economic* or pharmacoeconomi* or price* or "pricing":ti,ab,kw (Word variations have been searched) | 30849 |
| #18 | Economics or Economic* or Economic* or Cost or cost or "costs and cost analysis" or "costs and cost analysis" or "Cost allocation" or "Cost-allocation" or "Cost-benefit analysis" or "Cost control" or "Cost savings" or "Cost of illness" or "Cost sharing" or "deductibles and coinsurance" or "Medical savings accounts" or "Health care costs" or "Direct service costs" or "Drug costs" or "Employer health costs" or "Hospital costs" or "Health expenditures" or "Capital expenditures" or "Value of life" or "fees and charges" or charg* or fees or budget or budget or "fiscal" or fund* or financ* or "cost estimate" or "cost-estimate" or "cost variable" or "cost-variable" or "variable cost" or "variable-cost" or "unit cost" or "unit-cost" or pharmacoeconomic or "pharmaco economic" or "pharmaco-economic" or pric* or cost- effectiv* or cost effectiv* or cost-effectiv* or "cost effectiveness" or "cost-effectiveness" or "Socioeconomic" or "Cost utility" or "cost minimization" or "cost-minimization" or "health care utilisation" or "economic aspect" or "financial management" or "health economics" or reimburse* or re-imburse*:ti,ab,kw (Word variations have been searched) | 90606 |
| #19 | #12 or #15 or #16 or #17 or #18 | 94052 |
| #20 | #11 and #19 | 3 |
| #21 | Quality of life or "HRQOL" or "QOL" or "HRQL" or "health related quality of life" or "health utilities index" or "HUI" | 67424 |
| #22 | #11 and #21 | 20 |
| #23 | resource use or resource utili* or "resource usage" or "nursing cost" or "resource allocation" or "resource management" or "Health Care" or Health Care cost* or "direct cost" or "indirect cost" or "Economic burden" or "economic impact" or "Disease Burden" or "Burden of illness" or "Burden of sickness" or "Sickness Burden" or "burden of disease" or productivit* or "productivity" or "work day" or "working days" or "length of stay" or "duration of stay" or "extended stay" or "prolonged stay" or "duration of stay" or "prolonged stay" or "duration of hospitalisation" or "bed-days" or "bed days" or re-admi* or readmi* or "readmission" or "hospital readmission" or "ICU stay" or "ICU day" or absent* or "absenteeism" or "presenteeism" or "work day" or "working days" or "Lost Work productivity":ti,ab,kw (Word variations have been searched) | 76491 |
| #24 | #11 and #23 | 5 |
| #25 | Incidenc* or "Prevalence" or epidem* or mortalit* or natural histor* or demograph* or morbid* or risk or survival or etiology or aetiology or distribution or Frequency or pattern:ti,ab,kw (Word variations have been searched) | 397501 |
| #26 | #19 or #21 or #23 or #25 | 497830 |
| #27 | #1 and #26 | 64 |

The registries detailed in Table 2 were similarly searched for relevant trials/studies using various search terms.

Table 2: Registries included in the literature search

| **Database** | **Platform** | **Search strategy** |
| --- | --- | --- |
| US NIH registry & results database | <https://clinicaltrials.gov> | Advanced search / Search terms: Hereditary transthyretin amyloidosis, hATTR, polyneuropathy, cardiac amyloidosis, familial amyloid cardiomyopathy, familial amyloid polyneuropathy, Inotersen |
| WHO ICTRP registry | <http://apps.who.int/trialsearch/> | Advanced search / Search terms: Hereditary transthyretin amyloidosis, hATTR, polyneuropathy, cardiac amyloidosis, familial amyloid cardiomyopathy, familial amyloid polyneuropathy, Inotersen |
| CEA-registry | <http://healtheconomics.tuftsmedicalcenter.org/cear4/SearchingtheCEARegistry/SearchtheCEARegistry.aspx> | Search terms: Hereditary transthyretin amyloidosis, hATTR, polyneuropathy, cardiac amyloidosis, familial amyloid cardiomyopathy, familial amyloid polyneuropathy, Inotersen |

Abbreviations: CEA, Cost-effectiveness analysis; ICTRP, International Clinical Trials Registry Platform; NIH, National Institutes of Health; US, United States; WHO, World Health Organization.

## Congress Searches and Ad-hoc Searches

Abstract titles were searched using the keywords mentioned in Table 3 to identify relevant abstracts from the listed congresses. The searches were conducted on February 5^th^ 2018. The websites listed in Table 4 were also searched.

Table 3: List of congresses included in the literature search (2015-2017)

| **Research meeting** | **Abstract source** | **Search terms** |
| --- | --- | --- |
| European congress of hereditary ATTR amyloidosis & ATTR Amyloidosis meeting for patients and doctors (2015, 2017) | <https://www.attr-meeting.com> | Hereditary transthyretin amyloidosis, hATTR, polyneuropathy, cardiac amyloidosis, familial amyloid cardiomyopathy, familial amyloid polyneuropathy, Inotersen |
| International symposium on amyloidosis (2016) | <http://www.amyloidosis.nl/> | Same as above |
| European Academy of Neurology (2015, 2016, 2017) | <https://www.ean.org/> | Same as above |
| American Academy of Neurology (2015, 2016, 2017) | <https://www.aan.com/> | Same as above |
| International Society for Pharmacoeconomics and Outcomes Research US and Europe (2015, 2016, 2017) | <https://www.ispor.org/> | Same as above |
| American Association of Neuromuscular & Electrodiagnostic Medicine (2015, 2016, 2017) | <http://www.aanem.org/Home> | Same as above |
| Peripheral Nerve Society (2015, 2017) | <https://www.pnsociety.com/i4a/pages/index.cfm?pageid=1> | Same as above |
| American Neurological Association (2015, 2016, 2017) | <https://myana.org/> | Same as above |
| American College of Cardiology (2015, 2016, 2017) | <http://www.acc.org/#sort=%40fcommonsortdate86069%20descending> | Same as above |
| Heart Failure Society of America (2015, 2016, 2017) | <http://meeting.hfsa.org> | Same as above |
| European Society of Cardiology (2015, 2016, 2017) | <https://www.escardio.org/The-ESC> | Same as above |

Abbreviations: ATTR, Transthyretin-related amyloidosis

Table 4: Website searches

| **Database** | **Search field** | **Search terms** |
| --- | --- | --- |
| NICE | <https://www.nice.org.uk/> | Hereditary transthyretin amyloidosis, hATTR, polyneuropathy, cardiac amyloidosis, familial amyloid cardiomyopathy, familial amyloid polyneuropathy, Inotersen |
| RePEc website | <http://repec.org/> | Same as above |
| EQ-5D website | <https://euroqol.org/> | Same as above |
| The University of Sheffield’s ScHARRHUD database of health utilities’ evidence | <https://www.scharrhud.org/> | Same as above |
| HERC-maintained mapping algorithm database | <https://www.herc.ox.ac.uk/downloads/herc-database-of-mapping-studies> | Same as above |

Abbreviations: EQ-5D, HERC, Health Economics Research Centre; NICE, National Institute for Health and Care Excellence; RePEc, Research Papers in Economics
